# Fear conditioning prompts sparser representations of conditioned threat in primary visual cortex

**DOI:** 10.1093/scan/nsaa122

**Published:** 2020-09-09

**Authors:** Siyang Yin, Ke Bo, Yuelu Liu, Nina Thigpen, Andreas Keil, Mingzhou Ding

**Affiliations:** J. Crayton Pruitt Family Department of Biomedical Engineering, University of Florida, Gainesville, FL 32611, USA; J. Crayton Pruitt Family Department of Biomedical Engineering, University of Florida, Gainesville, FL 32611, USA; Center for Mind and Brain, University of California, Davis, CA 95618, USA; Department of Psychology, University of Florida, Gainesville, FL 32611, USA; Department of Psychology, University of Florida, Gainesville, FL 32611, USA; J. Crayton Pruitt Family Department of Biomedical Engineering, University of Florida, Gainesville, FL 32611, USA

**Keywords:** fear conditioning, alpha oscillations, visual representation, sparsification, attention

## Abstract

Repeated exposure to threatening stimuli alters sensory responses. We investigated the underlying neural mechanism by re-analyzing previously published simultaneous electroencephalogram-functional magnetic resonance imaging (EEG-fMRI) data from humans viewing oriented gratings during Pavlovian fear conditioning. In acquisition, one grating (CS+) was paired with a noxious noise, the unconditioned stimulus (US). The other grating (CS-) was never paired with the US. In habituation, which preceded acquisition, and in extinction, the same two gratings were presented without US. Using fMRI multivoxel patterns in primary visual cortex during habituation as reference, we found that during acquisition, aversive learning selectively prompted systematic changes in multivoxel patterns evoked by CS+. Specifically, CS+ evoked voxel patterns in V1 became sparser as aversive learning progressed, and the sparsified pattern appeared to be preserved in extinction. Concomitant with the voxel pattern changes, occipital alpha oscillations were increasingly more desynchronized during CS+ (but not CS-) trials. Across acquisition trials, the rate of change in CS+-related alpha desynchronization was correlated with the rate of change in multivoxel pattern representations of CS+. Furthermore, alpha oscillations co-varied with blood-oxygen-level-dependent (BOLD) data in the ventral attention network, but not with BOLD in the amygdala. Thus, fear conditioning prompts persistent sparsification of voxel patterns evoked by threat, likely mediated by attention-related mechanisms

## Introduction

Accurate detection and evaluation of threat and danger is crucial to survival. The mammalian brain has evolved mechanisms that bias perceptual systems towards sensory cues that predict aversive outcomes (Pessoa and Adolphs, 2010). For example, neurons in human primary visual cortex (V1) alter their tuning properties to selectively amplify visual threat cues (Miskovic and Keil, 2012). Across species, sensory neurons in rodents also undergo selective plasticity to better represent threat cues, both in the visual cortex (Shuler and Bear, 2006) and in the auditory cortex (Weinberger, 2004). These observations suggest that associative learning of contingencies between a conditioned visual stimulus (CS+) and an aversive unconditioned stimulus (US) prompts changes in the sensory neural representation of CS+.

Paralleling conditioned auditory receptive field plasticity in rats (Headley and Weinberger, 2011), sensory changes in response to aversive conditioning can be characterized as selectively heightened population gain for the critical CS+ feature (Li *et al.*, 2019). For example, if CS+ and CS- differ in orientation, a plasticity-based view predicts that differential aversive conditioning selectively biases orientation tuning of visuocortical neurons to optimize population coding for the CS+ (Miskovic and Keil, 2012; Miskovic and Anderson, 2018). A growing body of work has converged to support this hypothesis (McTeague *et al.*, 2015; Antov *et al.*, 2020). What is not known, however, is how such changes in visuocortical tuning are implemented in visual cortex as aversive learning progresses. Possible hypotheses include (1) an increase in visuocortical population activity when viewing a threat-associated cue (Morris *et al.*, 1998a; Phelps *et al.*, 2006) and (2) the emergence of highly connected visuocortical networks that provide efficient and sparse coding of CS+ features, through Hebbian mechanisms (Simoncelli and Olshausen, 2001; Miskovic and Keil, 2012; Headley and Weinberger, 2013). Testing of these competing views in human observers has been elusive. This is partly due to the interpretational ambiguity of scalp recorded electrophysiological signals, which typically rely on trial averaging. Trial averaged responses increase with the amount of activated neural tissue, but they can also increase with growing phase similarity across trials (e.g. Moratti *et al.*, 2007). Efficiently operating sparse networks are predicted to produce temporally precise evoked mass responses that are similar across trials (Keil *et al.*, 2007). Thus, evoked responses such as the early visual evoked potential may increase in amplitude as a result of overall increased neural activity or as a result of the formation of sparse networks yielding temporally precise activation. In terms of fMRI, sparsification in neural representation prompted by aversive experience would be reflected in increasingly altered voxel patterns for CS+, but not for CS-, during fear conditioning, characterized by decreasing numbers of voxels contributing to the representation of the CS+. Heightened population responses when viewing the CS+ would, however, result in heightened BOLD in a larger number of voxels. Thus, we addressed the two competing theoretical notions of learning-related population activity increase versus learning-related sparsification in neural representation by re-analyzing EEG-fMRI data from a previous published study (Yin *et al.*, 2018) and quantifying the evolution of fMRI patterns evoked by conditioned stimuli.

Recent work shows that autonomic orienting responses (e.g. heart rate [HR]) to conditioned threat attenuate, along with CS+-related response in the limbic structures, as aversive learning progresses (Yin *et al.*, 2018). To what extent this process is accompanied by enhanced attentional orienting is not clear. We measured EEG concurrently with BOLD so that we could use EEG alpha band activity (8–12 Hz) as an index of visual attention engagement with the conditioned stimuli. Transient suppression of spectral power in the alpha band (i.e. event-related desynchronization or ERD) has been taken to index attentive engagement of visual cortex in processing task-relevant stimuli (Klimesch, 2012; Zumer *et al.*, 2014), and the more task-relevant the stimuli, the stronger the alpha ERD (Klimesch *et al.*, 2011; Auksztulewicz *et al.*, 2017). We hypothesized that as threat cues acquire increased task-relevance through conditioning, alpha power would show greater ERD after CS+ stimuli compared to CS- stimuli, reflecting enhanced CS+-related attentional orienting, and this effect would become stronger as learning progressed.

BOLD responses in V1 and visual alpha oscillations can both be modulated by attention control networks (Posner and Gilbert, 1999; van Diepen *et al.*, 2016). Alpha power reductions index target selection during a range of selective attention tasks (e.g. Rohenkohl and Nobre, 2011). However, in fear conditioning, the higher-order structures contributing to the selective visuocortical changes remain unclear. Two potential sources of modulatory bias signals are the ventral attention network (VAN) and limbic emotion-modulated circuits centered around the amygdala (Yates *et al.*, 2010; McHugo *et al.*, 2013). The VAN, including right temporal-parietal junction (rTPJ) and right ventrolateral prefrontal cortex (rVLPFC), is involved in directing attention toward salient stimuli (Corbetta and Shulman, 2002; Armony and Dolan, 2002), whereas the amygdala encodes information about the motivational significance of sensory input (Amaral *et al.*, 2003; Paton *et al.*, 2006) and may modulate visual cortex through connections with the basal forebrain (Peck *et al.*, 2014; Allen *et al.*, 1998) or with parietal and temporal cortex (Amaral *et al.*, 2003; Keil *et al.*, 2009). We examined these competing possibilities by correlating alpha power fluctuations with fMRI from the VAN and the amygdala.

## Materials and methods

This study is a reanalysis of previously published data (Yin *et al.*, 2018). The previous study by Yin *et al.* (2018) was motivated by the observation that selective amygdala activation by conditioned threat is often not found in human imaging studies and proceeded to test the amygdala adaptation hypothesis. The present paper focused on activities in visual cortex and examined the neural basis of previously reported changes with aversive learning in visual responses to conditioned fear.

### Experimental procedure

#### Participants.

The experimental protocol was approved by the Institutional Review Board of the University of Florida. Eighteen healthy college students (aged 17–33 years, 9 females) provided written informed consent and participated in the study. The participants were either paid or given course credits in accordance with Institutional Review Board guidelines.

#### Stimuli.

**Fig. 1.**
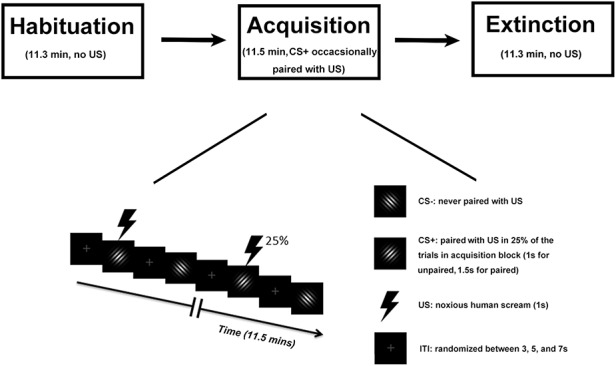
Experimental paradigm. Top: Temporal order of the three blocks. Bottom: timeline and stimuli used during the acquisition block. For the habituation block and the extinction block, the two Gabor patches and inter-trial interval (ITI) were the same, except that no US was presented.

Two Gabor patches (sine wave gratings filtered with a Gaussian envelope, Michelson contrast = 1) with the same spatial frequency (1.5 cycles/degree), differing only in orientation (45° and 135°), were designated as CS+ and CS-, respectively; they were not counterbalanced across subjects (See Figure 1). Both stimuli were projected onto a back-illuminated screen (60 cm × 60 cm) placed 230 cm away from the participant’s head and viewed through a set of prismatic glasses attached to the radio frequency head coil. The US was a 1-s human scream delivered by an MRI compatible headphone at around 95 dB. For CS- trials and CS+ trials where CS+ and US were not paired, the Gabor patches were shown for 1 s. For CS+ trials where CS+ and US were paired, the US started 0.5 s following CS+ onset and co-terminated 1 s later. This approach ensured that CS+ and CS- trials included in the analysis did not differ and that paired CS+ trials had a sufficient lead time as well as co-termination with the US as required for classical (delay) conditioning.

#### Paradigm.

The experiment consisted of three blocks: habituation, acquisition and extinction (Figure 1). Each block comprised 120 trials and lasted about 12 min. During the acquisition block, one Gabor patch was designated as CS+ and the other as CS-. In the habituation block, which preceded the acquisition block, the two Gabor patches occurred with equal probability in a pseudo-random order, determined by a procedure in which the two Gabor patches were randomly (toss of a fair coin) drawn from two pools without replacement, under the constraint that not more than two CSs of one kind (future CS+, future CS-) appeared in direct sequence, as is typical in fear conditioning work (Lonsdorf *et al.,* 2017; Auksztulewicz *et al.*, 2017). During acquisition, the same pseudo-randomization was again applied to result in a different order with the same constraints as described for habituation. In addition, acquisition always started with a CS+ trial, and the first four CS+ stimuli were always paired with the US to facilitate contingency learning. Subsequently, 25% of CS+ stimuli were paired with the US. CS- stimuli were never paired with the US. For analysis, paired CS+ trials were not included due to fMRI contamination by US evoked responses. For notational simplicity, in what follows, we use the term CS+ trials when referring to unpaired CS+ trials in which no US occurred. In the extinction block, which followed the acquisition block, the stimuli and procedure were the same as the habituation block, i.e. the pseudo-randomization procedure was again applied to result in a pseudo-random order with the constraint that no more than two CSs of the same type appeared in a row. We note that, within a given block, the order of trials was the same for each subject to facilitate trial-by-trial averaging across subjects, which is essential for analyzing the temporal dynamics of conditioning across trials at a population level (Yin *et al.*, 2018). For each of the three blocks, the inter-trial interval (ITI) was randomized between 3, 5 and 7 s (see Figure 1).

### Data acquisition

#### fMRI  data.

fMRI images were acquired on a 3-Tesla Philips Achieva whole-body MRI system (Philips Medical Systems, Netherlands) using a T2*-weighted echoplanar imaging sequence (echo time (TE) = 30 ms; repetition time (TR) = 1980 ms; flip angle = 80°). Each whole-brain volume consisted of 36 axial slices (field of view: 224 mm; matrix size: 64 × 64; slice thickness: 3.50 mm; voxel size: 3.5 × 3.5 × 3.5 mm). A T1-weighted high-resolution structural image was also obtained from each participant. For one subject, the fMRI data during habituation were not properly saved to the disk, and the data from 17 subjects were thus used for fMRI analysis for the habituation block. For all other analyses, fMRI data from all 18 subjects were used.

#### EEG data.

EEG data was recorded simultaneously with fMRI using a 32-channel MR-compatible EEG system (Brain Products GmbH, Germany). Thirty-one sintered Ag/AgCl electrodes were placed according to the 10–20 system with the reference channel being FCz during recording. One additional electrode was placed on the participant’s upper back to monitor the electrocardiogram (ECG). ECG data were used to enable HR analysis and to aid in the removal of the cardioballistic artifacts. The impedance from all scalp channels was kept below 10 kΩ during the entire recording session as recommended by the manufacturer. The online band-pass filter had cutoff frequencies at 0.1 and 250 Hz. The filtered EEG signal was then sampled at 5 kHz and digitized to 16-bit. The EEG recording system was synchronized with the scanner’s internal clock, which, along with the high sampling rate, was essential to ensure the removal of the MRI gradient artifacts.

### Regions of interest

**Fig. 2.**
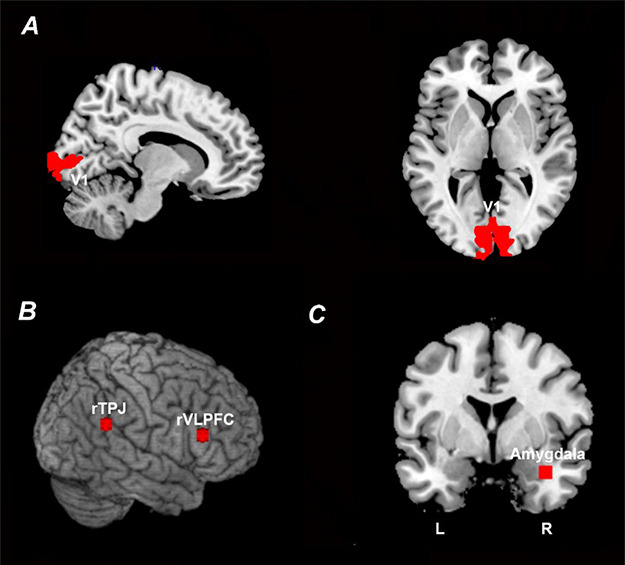
ROI definition. (A) V1 ROI defined according to a recently published retinotopic atlas of the visual cortex by Wang *et al.* (2015). (B) rTPJ and rVLPFC ROIs defined according to previously published coordinates (Geng and Vossel, 2013; Yin *et al.*, 2018). (C) Right amygdala ROI defined according to previously published coordinates based on US activation (Yin *et al.*, 2018).

Four regions of interest (ROIs) were considered: the primary visual cortex or V1 (Figure 2A), the rTPJ and the rVLPFC, both of the VAN (Figure 2B) and the right amygdala (Figure 2C). The V1 ROI was bilateral and defined using a recently published template of retinotopic regions of the visual cortex (Wang *et al.*, 2015); this ROI contained 473 contiguous voxels. The rTPJ and rVLPFC ROIs were defined to be 6 mm spheres centered at the previously published coordinates of rTPJ (Geng and Vossel, 2013) and rVLPFC (Yin *et al.*, 2018); they each contained 33 voxels. The right amygdala ROI was chosen to be a 6 mm sphere centered at the previously determined peak-activation voxel from contrasting US against CS- (Yin *et al.*, 2018); this ROI contained 33 voxels. The left amygdala was not activated in this contrast and thus not considered further.

### Data processing.

#### fMRI data preprocessing.

All fMRI data analyses were performed in Statistical Parametric Mapping (SPM) (http://www.fil.ion.ucl.ac.uk/spm/). Preprocessing steps included slice timing, motion correction, and normalization to the Montreal Neurological Institute template. Normalized images were spatially-smoothed with a 7 mm full width at half maximum Gaussian kernel. This spatial smoothing step was omitted for the representational similarity analysis (RSA) to better preserve spatial patterns. The BOLD time series were high-pass filtered with a cutoff frequency at 1/128 Hz.

#### EEG data preprocessing.

There are two major sources of MRI-related artifacts in EEG that are recorded simultaneously with fMRI: the gradient artifacts and the cardioballistic artifacts. Gradient artifacts were removed by subtracting an average artifact template from the data set as implemented in Brain Vision Analyzer 2.0 (Brain Products GmbH, Germany). The artifact template was constructed by using a sliding-window approach which involved averaging the EEG signal across the nearest 41 consecutive volumes. The cardioballistic artifacts were also removed by an average artifact subtraction method (Allen *et al.*, 1998). In this method, the R peaks were first detected in the ECG recordings by the algorithm in Brain Vision Analyzer, and then visually inspected to ensure accuracy. The appropriately detected R peaks were utilized to construct a delayed average artifact template over 21 consecutive heartbeat events. The cardioballistic artifacts were then removed by subtracting the average artifact templates from the EEG data. After these two steps, the EEG data were band-pass filtered between 0.5 and 50 Hz, down-sampled to 250 Hz, re-referenced to the average reference (Nunez *et al.*, 1997) and exported to EEGLAB (Delorme and Makeig, 2004) for analysis.

#### HR analysis.

The time between heart beats (RR interval) was estimated from the ECG data and transformed into instantaneous HR (inverse of RR interval). The time range from 1-s prestimulus to 5-s post-stimulus was divided into 1-s bins, and each instantaneous HR was weighted proportionally to the fraction of the bin it occupied (Gatchel and Lang, 1973; Graham, 1980) to yield stimulus-locked HR change times series within a trial. This single-trial stimulus-locked HR change time series was then averaged across all trials within a block to assess how, on average, CS+ and CS- affected stimulus-locked HR changes in habituation, acquisition and extinction.

In addition, for each of the three blocks, the trial-by-trial relative HR change (deceleration) was estimated by taking the stimulus-locked HR change in the interval (0.5 s, 1.5 s) from each trial. Because single-trial HR data are noisy, it was necessary to apply smoothing to the resulting time series (one HR change value for each trial). Specifically, the trial-by-trial time series for CS+ and CS- trials were separately smoothed using a moving window (Gaussian kernel, bandwidth = 12). This resulted in two smoothed time series, one for the CS+ and one for the CS-. For illustration and correlation analyses, we computed the trial-wise CS+/CS- difference in HR deceleration by subtracting the CS+ HR time series from the CS- HR time series.

#### Single-trial estimation of BOLD response.

The BOLD response was estimated on a trial-by-trial basis using the beta series method (Rissman *et al.*, 2004, 2008). In this method, every stimulus was associated with a separate regressor in the general linear model. Rigid body movements were included as regressors of no interest. Solving the general linear model yielded a beta value for each trial in each voxel. We conducted this analysis separately for each of the three blocks (Habituation, acquisition and extinction) and obtained, for each block, 120 single-trial beta values corresponding to the CS+ and CS- trials (Yin *et al.*, 2018). Except for the 18 CS+ trials that were paired with the US in acquisition, the remaining single-trial beta values were subjected to RSA, pattern sparsity and alpha-ERD correlation analysis.

#### Representational similarity analysis.

Multivoxel representations of CS+ and CS- can be studied using RSA (Visser *et al.*, 2011, 2013). To maximally retain information at a finer spatial scale (Dunsmoor *et al.*, 2014), we applied the beta series method to the BOLD time series prior to spatial smoothing to obtain single-trial beta values. For a given ROI, a vector was created from the beta values of all the voxels to represent the spatial pattern in response to a single presentation of a stimulus; the length of the vector equaled the number of voxels in that ROI. Reference representations of CS+ and CS- for the V1 ROI were generated from averaging the single-trial multivoxel patterns across all the trials (60 each) in the habituation block. During acquisition and extinction, to generate the time course of neural representational changes over trials (i.e. similarity curve), a sliding window approach was adopted, in which the time window used was five trials in duration and the step size was one trial. After the moving average (five-trial average), each trial-averaged vector in acquisition and extinction was correlated with its reference representation derived from habituation to assess pattern similarity. The correlation coefficients were Fisher-z transformed, averaged across participants, re-transformed back to correlation coefficients and plotted as a function of time-on-task to yield the time course of changes in neural representations of CS+ and CS-. The slope of the time course was estimated by linear fit for each individual subject’s similarity curve and taken as a measure of the rate of change in neural representations for that subject. A paired *t*-test was used to compare the slopes between CS+ and CS- across participants. It is worth noting that RSA is not a machine learning technique and does not involve the splitting of data into training data and testing data (Bach *et al.*, 2011).

#### Pattern sparsity analysis.

To investigate the changes in stimulus-evoked BOLD patterns vis-à-vis the changes in stimulus-evoked BOLD magnitude during acquisition, we quantified the change in sparsity of the voxels in the representational pattern evoked by CS+ and CS-. First, to assess the broad temporal change across acquisition, we divided acquisition into an early time period (*t* < 5.6 mins) and a late time period (*t* > 5.6 mins). Second, for the stimulus type (CS+ or CS-) showing significant pattern change relative to habituation, we counted the voxels that represented this stimulus type (i.e. representational voxels) for each time period. A voxel entered this count only if it met all of the following three requirements: (1) It showed larger average activity across trials for this stimulus type (e.g. CS+) than the other type (e.g. CS-) where the average activity was defined as the mean of single-trial betas, (2) the average activity for the stimulus type from the voxel was greater than the average activity from all the voxels within the ROI and (3) the standard deviation of the activity across trials from the voxel was less than the mean of the standard deviation from all the voxels within the ROI. Thus, a representational voxel defined this way was a voxel that was selectively, strongly and consistently enhanced for a given stimulus type across trials, and over neighboring voxels. Finally, the number of representational voxels and the averaged betas within these voxels were compared between early and late period of acquisition to assess the changes in stimulus-evoked representational patterns and in stimulus-evoked BOLD magnitude. The same analysis was also applied to the habituation block and the extinction block for comparison and for examining whether the sparsified neural representations of conditioned threat persisted over the extinction phase of the experiment.

#### EEG alpha ERD estimation.

ERD of posterior alpha oscillations (8 to 12 Hz) was taken as an indicator of visual activation and attention orienting. Alpha ERD was estimated for each trial as follows. First, the EEG signal was epoched from −1 s to 2 s with 0 s denoting the onset of CS+ or CS-. Second, the EEG signal within each epoch was divided into overlapping moving windows with 200 ms in duration and 20 ms in step size. Third, the EEG data in each window was zero-padded to 5 times its original length (250 points after padding) to enhance spectral resolution from 5 Hz to 1 Hz. Fourth, the EEG power spectrum for each window was calculated using a nonparametric multi-taper approach with 3 tapers (Mitra and Pesaran, 1999), and the alpha power was estimated by averaging the power spectrum between 8 and 12 Hz. The baseline was defined as the alpha power within the window centered at stimulus onset across all trials. The single-trial alpha ERD was calculated by subtracting baseline alpha power from the alpha power within each moving window and dividing the differenceby baseline alpha power. For a given post-stimulus time window, alpha ERD could be plotted as a function of acquisition trials, and the slope of this function obtained from a linear regression analysis provided a rate of change of alpha ERD, which was taken as a measure of change in visual attention engagement. A paired *t*-test was used to compare the slopes between CS+ and CS- category across participants. It is worth noting that the present experimental paradigm lacks an explicit attention manipulation. Using alpha ERD as an index of visual attention engagement is indirect and relies on assumptions derived from prior research (Klimesch *et al.*, 2011; Auksztulewicz *et al.*, 2017).

#### Alpha-BOLD correlation.

To assess which regions of the brain (rTPJ, rVLPFC or right amygdala) modulated alpha power, two analyses were carried out for the acquisition block: across-participant correlation analysis and across-trial correlation analysis. Across-participant correlation was computed as the correlation coefficient between differential occipital alpha ERD (CS+ minus CS-) averaged across trials within the acquisition block and differential beta values from rTPJ, rVLPFC or right amygdala (CS+ minus CS-) averaged across trials within the acquisition block. Across-trial correlation was assessed by correlating the single-trial alpha ERD averaged across participants and single-trial BOLD beta values averaged across participants. We sought converging evidence by performing these two types of analyses.

## Results

### HR changes

**Fig. 3.**
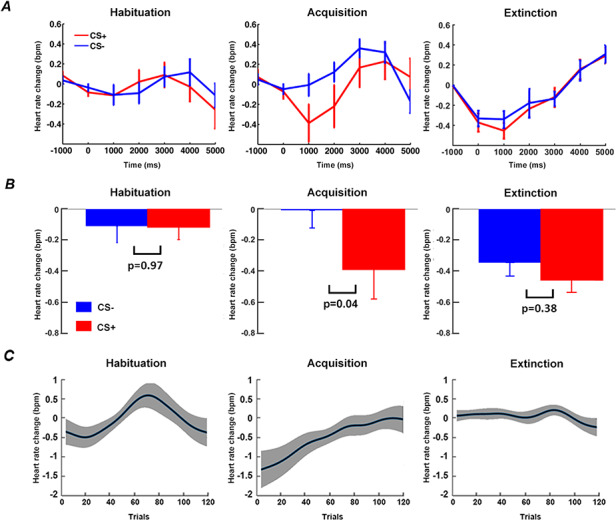
Heart rate (HR) analysis. (A) Event-related HR changes during habituation, acquisition and extinction. (B) Statistical comparison of HR between CS+ and CS- at time = 1 s (0.5 s to 1.5 s). (C) Time course of relative event-related HR changes (CS+ minus CS-) over trials in habituation, acquisition, and extinction. Note: Figure 3A (left and middle), 3B (left and middle) and 3C (left and middle) are adapted from Yin *et al.* (2018) under CC BY 4.0 and included here for comparison with Figure 3A (right), 3B (right) and 3C (right).

As shown in Figure 3A and 3B, in both habituation and extinction, the average event-related HR changes did not differ between CS+ and CS-. During acquisition, greater HR deceleration was observed following CS+ compared to CS-, demonstrating that participants acquired the contingencies of the experiment, and exhibited defensive orienting to the CS+. Figure 3C shows the time course of relative event-related HR change (CS+ minus CS-) over trials for the three blocks. During habituation, as expected, there was no systematic trend in the differential HR time course across trials. For acquisition, greater CS+-related HR deceleration was apparent in the early part of the block, and the difference gradually diminished as learning progressed and disappeared toward the end of the block (Yin *et al.*, 2018). There was no systematic trend in event-related HR change between CS+ and CS- over the entire extinction block.

### Dynamic changes of neural representations of CS+ in acquisition

**Fig. 4.**
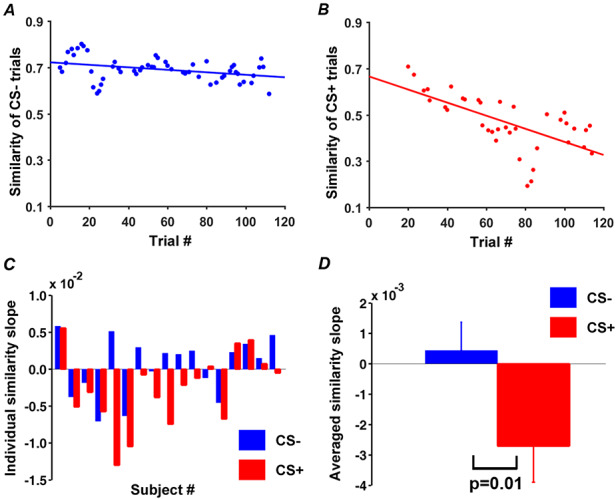
Pattern similarity changes during acquisition in V1. (A) Time course of pattern similarity change in V1 for CS- trials (Subject 8 in (C)). (B) Time course of pattern similarity change in V1 for CS+ trials from the same subject. (C) Slopes of linear fits to pattern similarity curves such as the ones in (A) and (B) for each participant. (D) Slopes of similarity curves between CS+ and CS- in V1 were significantly different.

Reference representations for CS+ and CS- in V1 were obtained by averaging single-trial BOLD responses to CS+ and CS- across habituation trials. Applying the moving window approach to acquisition (window size: five trials; step size: one trial), CS+ and CS- evoked patterns in V1 in each moving window were correlated with their respective reference representational patterns (see Methods: ‘Representational similarity analysis’), to yield the time courses of RSA pattern similarity changes (similarity curves); see Figure 4A and 4B for example similarity curves from an individual subject. Across participants, the RSA similarity curve for CS+ showed a decreasing trend, while the similarity curve for CS- varied unsystematically, resulting in a flat average slope (Figure 4C and 4D). This demonstrates that the patterns evoked by CS+ during acquisition were systematically departing from its reference representation pattern, whereas the CS- evoked patterns did not exhibit any systematic change. The rate of pattern similarity change for each individual, indexed by the slope of the linear fit to the similarity curve, is shown in Figure 4C. Across participants, as shown in Figure 4D, the slopes of CS+ RSA similarity curves were significantly different from the slopes of CS- RSA similarity curves (*P* = 0.01, d = 0.85).

### Changes in pattern sparsity during acquisition

**Fig. 5.**
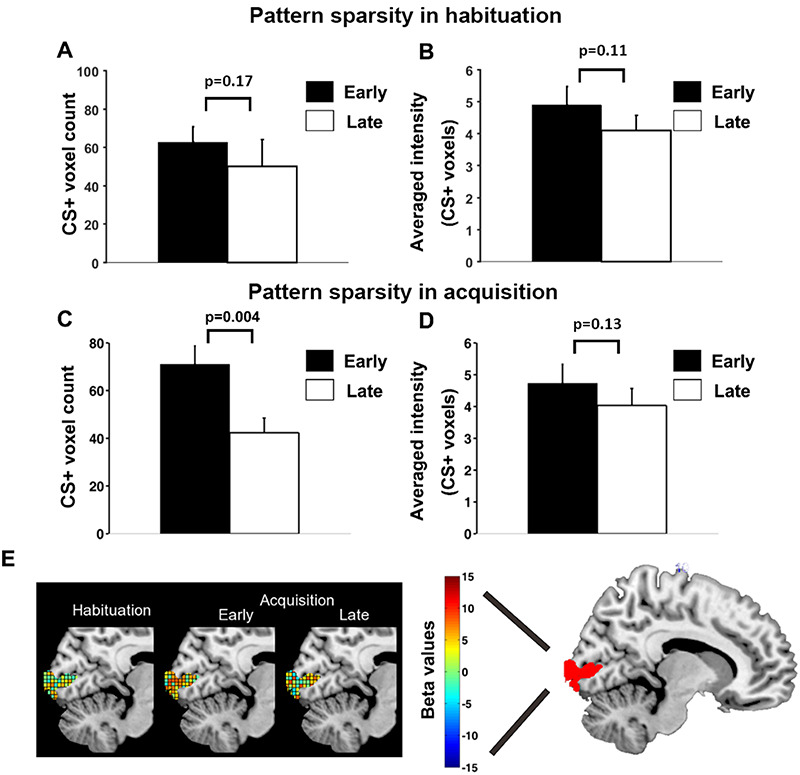
Pattern sparsity analysis for CS+ trials. (A) No significant difference in number of representational voxels for CS+ in V1 between early and late habituation. (B) No significant difference in average BOLD activation between early and late habituation. (C) Number of representational voxels for CS+ in V1 was significantly lower in late acquisition than early acquisition. (D) No significant difference in average BOLD activation between early and late acquisition. (E) Schematic illustration of increasing sparsity observed during CS+ trials over time: CS+ evoked multivoxel patterns of beta values in habituation, early acquisition and late acquisition.

To more closely examine the acquisition-related CS+ pattern changes over time and whether the changes were specific to acquisition, we divided the habituation block and the acquisition block into an early period and a late period (the extinction block was similarly examined; see later). For each time period, the V1 representational voxels (see Methods: ‘Pattern sparsity analysis’) for CS+ were counted and shown in Figure 5A for habituation and Figure 5C for acquisition, and the averaged betas within these voxels, representing average BOLD activation evoked by CS+ for the ROI, were calculated and plotted in Figure 5B for habituation and Figure 5D for acquisition. For habituation, the number of representational voxels for CS+ was not significantly different between the early and the late period (*P* = 0.17, d = 0.26) (Figure 5A), whereas the average CS+-evoked BOLD response magnitude was also not significantly different between the two periods (*P* = 0.11, d = 0.33) (Figure 5B). For acquisition, the number of representational voxels for CS+ was significantly lower in the late period relative to the early period (*P* = 0.004, d = 0.97) (Figure 5C), while the average CS+-evoked BOLD response magnitude did not undergo significant change from early to late period (*P* = 0.13, d = 0.29) (Figure 5D). Figure 5E illustrates schematically the multivoxel patterns evoked by CS+ with the color of each cube (i.e. voxel) reflecting the beta value of that voxel; in the late period of acquisition, CS+ was represented by fewer voxels compared to the early period of acquisition.

### EEG alpha-band activity

**Fig. 6.**
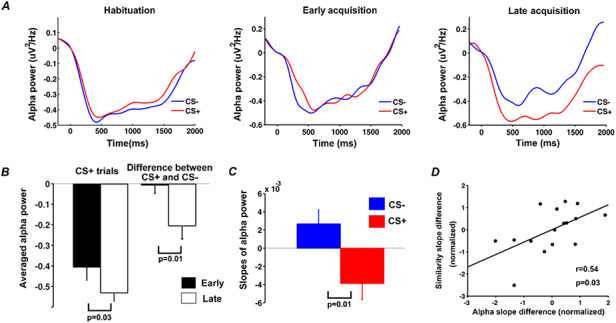
Event-related alpha desynchronization during habituation and acquisition. (A) Alpha-band (8–12 Hz) power averaged across CS+ trials and across CS- trials during habituation, the early period of acquisition and the late period of acquisition. (B) CS +-evoked alpha ERD and the difference in CS+ and CS- alpha-band power for early and late acquisition periods. (C) The slope of linear fit to the time course of alpha-band power across acquisition trials. (D) Relation between the rate of event-related alpha-band power decrease and the rate of pattern similarity change in V1 (each point in the plot represents one participant).

Stimulus-evoked time course of event-related alpha-band power (8 to 12 Hz) within a trial was shown in Figure 6A for habituation as well as for early and late periods of acquisition. Quantifying alpha ERD using average alpha power in the interval 600 to 1000 ms, there was no significant difference in alpha power between CS+ and CS- in habituation or in early period of acquisition, but alpha power was significantly lower following CS+ in late period of acquisition (*P* = 0.03, d = 0.67) (Figure 6B). In line with these findings, a paired *t*-test revealed greater differential (CS+ minus CS-) alpha ERD in the late compared to the early period of acquisition (*P* = 0.01, d = 0.85) (Figure 6B). To further quantify these cross-trial dynamics, we computed the time course of alpha ERD changes across trials using the moving window approach mentioned earlier (window size: five trials; step size: one trial) and estimated the slope of the linear fit to alpha ERD changes across CS+ trials and CS- trials. A Wilcoxon signed-rank test indicated that the resulting slopes differed significantly (*P* = 0.01, d = 0.85) (Figure 6C).

### Relation between alpha ERD change over trials and BOLD pattern similarity change over trials during acquisition

Exploring the relationship between across-trial changes in alpha ERD and BOLD pattern similarity changes in CS+ evoked patterns in V1 during acquisition, we observed a positive correlation at r = 0.52 (*P* = 0.03, d = 1.22) between the differential slope of alpha power ERD and the differential slope of pattern similarity curve (Figure 6D). This finding suggests that as aversive learning progressed, participants with more pronounced representational voxel pattern changes in V1 tended to show progressively stronger alpha ERD. It is worth noting that when assessing the number of representational voxels for CS+ as a function of trials using the same moving window approach, we found that the slope of such a sparsity change curve and the slope of the alpha ERD change curve was not significantly correlated at r = 0.22 (*P* = 0.4, d = 0.45).

### Alpha-BOLD correlation during acquisition

**Fig. 7.**
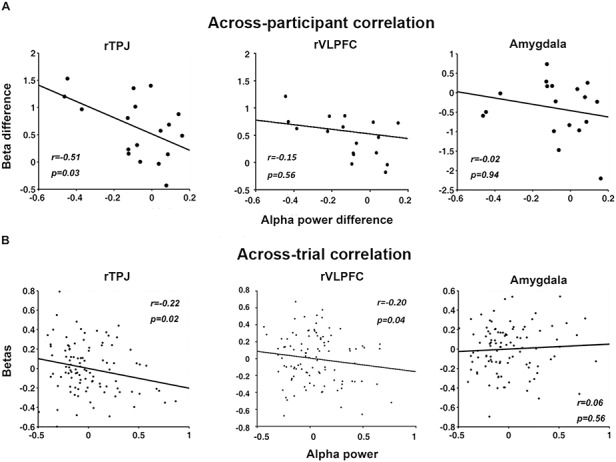
EEG-BOLD coupling in acquisition. (A) Across-participant correlations between alpha ERD and BOLD in the rTPJ and rVLPFC, both of the ventral attention network, and the right amygdala. A negative correlation was observed between alpha ERD difference (CS+ minus CS-) and the difference in rTPJ beta values (CS+ minus CS-). No correlation was observed between alpha ERD difference and the estimated beta difference in rVLPFC and right amygdala. Each point in the plots represents a participant. (B) Across-trial correlations between alpha ERD and BOLD in rTPJ, rVLPFC and right amygdala. There was a significant negative correlation between trial-wise alpha power and trial-wise beta value from rTPJ and rVLPFC, but no correlation between trial-wise alpha power and trial-wise beta from right amygdala. Each point in the plots represents a trial.

Concurrent recordings of EEG and fMRI afforded the opportunity to examine the sources of modulatory signals for alpha ERD. In acquisition, as shown in Figure 7, alpha power desynchronization was found to be significantly negatively correlated with the BOLD from rTPJ both across participants (*r *= −0.51, *P *= 0.03, d = −1.19) (Figure 7A left) and across trials (*r *= −0.22, *P *= 0.02, d = −0.45) (Figure 7B left). For rVLPFC, the same analysis showed that there was a significant across-trial correlation (r = −0.20, *P* = 0.04, d = −0.41) but not a significant across-participant correlation (r = −0.15, *P* = 0.56, d = −0.30). Neither across-participant nor across-trial correlations were found to be significant between alpha power desynchronization and BOLD in right amygdala (Figure 7A right and 7B right).

### Neural dynamics during extinction

**Fig. 8.**
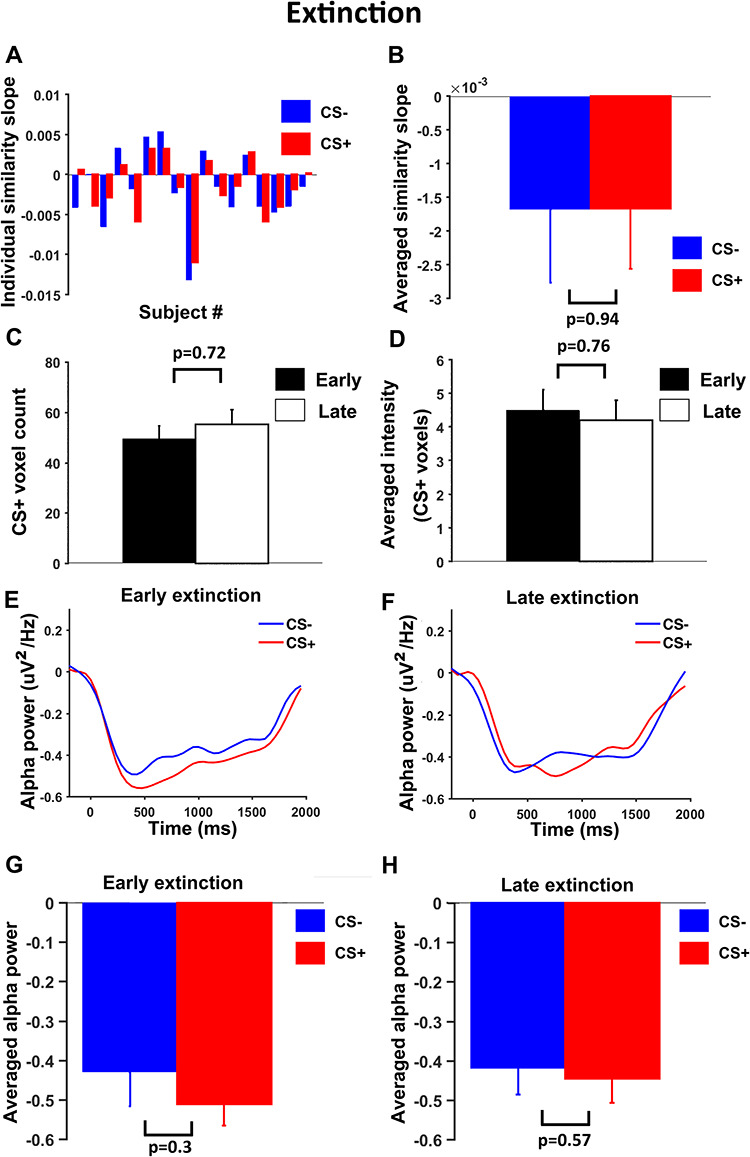
Neural dynamics in V1 during extinction. (A) Slopes of linear fits to pattern similarity curves for each participant. (B) Slopes of similarity curves were not significantly different between CS+ and CS-. (C) No significant difference in number of representational voxels for CS+ between early and late extinction. (D) No significant difference in average BOLD activation for CS+ between early and late extinction. (E) and (F) Alpha-band ERD for CS+ and CS- trials during early extinction and late extinction. (G) and (H) No significant difference in alpha ERD between CS+ and CS- in either early or late extinction.

We carried out a similar analysis for the extinction data. During extinction, as shown in Figure 8, slopes of pattern similarity change time course were not different between CS+ and CS- (*P* = 0.94, d = 0.025) (Figure 8A and 8B); the number of representational voxels for CS+ was not different between early and late period (*P* = 0.72, d = 0.03) (Figure 8C) and CS+ evoked average BOLD activation were also not different between early and late periods (*P* = 0.76, d = 0.11) (Figure 8D). Figure 8E and 8F showed the stimulus-evoked alpha ERD time courses within a trial for early and late extinction. Statistical comparisons revealed that alpha ERD for CS+ and CS- were not significantly different both during early (*P* = 0.3, d = 0.35) and late (*P* = 0.57, d = 0.19) periods of extinction.

### Additional analyses

First, a closer inspection of Figures 5C and 8C revealed that the number of representational voxels for CS+ during early extinction (53.0 ± 4.7) was not significantly different from the number of representational voxels for CS+ in late acquisition (42.5 ± 5.5) (*P* = 0.17, d = 0.47) but was marginally smaller than the number of representational voxels for CS+ in early acquisition (71.0 ± 7.7) (*P* = 0.067, d = 0.65). This suggested that the sparsified neural representations of CS+ reached at the end of acquisition might have persisted in extinction. Second, to formally test how interactions among experimental blocks reflected the change in the number of representational voxels for CS+, we performed a two-factor ANOVA, with one factor being the block (habituation, acquisition, extinction) and the other being the period within a block (early, late). The interaction effect was marginally significant (*P* = 0.055, d = 0.68). There was a significant early-to-late main effect (*P* = 0.02, d = 0.85), which was primarily driven by the reduction in the number of representational voxels for CS+ during acquisition (Figure 5C), and there was no significant main effect of block (*P* = 0.91, d = 0.04). Note that this ANOVA tests many additional differences not relevant to the present investigation, which focused on the temporal dynamics in acquisition, with a clear hypothesis of no CS+/CS- difference during habituation, followed by increased sparsity only for the CS+. Despite its omnibus characteristic, the present ANOVA results are consistent with the analyses earlier, finding strong support for these hypotheses. Third, in addition to primary visual cortex, the sparsity algorithm was also applied to the other ROIs considered in this work, including right amygdala, rTPJ and rVLPFC. We found that there was no significant difference in the number of representational voxels for CS+ between the early and the late period of acquisition in rTPJ and rVLPFC (rTPJ: *P* = 0.79, d = 0.07; rVLPFC: *P* = 0.69, d = 0.13) but a significant reduction from early to late acquisition in right amygdala (*P* = 0.03, d = 0.82). There was no significant early-to-late difference in average CS+ evoked BOLD activity in all three ROIs (amygdala: *P* = 0.51, d = 0.36; rTPJ: *P* = 0.85, d = 0.03; rVLPFC: *P* = 0.19, d = 0.63).

## Discussion

In classical fear conditioning, a neutral stimulus (CS+), through repeated association with an aversive stimulus (US), comes to elicit defensive responses in the absence of the original aversive stimulus. The sensory neural response to CS+ also undergoes systematic changes in this process. Here, we examined this problem by recording simultaneous EEG-fMRI from human participants performing a classic Pavlovian fear conditioning paradigm and found that: (1) in primary visual cortex (V1), the representational voxel pattern evoked by the CS+ became sparser as learning progressed, and this sparsification appeared to persist in extinction; (2) alpha ERD following CS+ (but not CS-) became more pronounced as learning progressed, suggesting heightened engagement of visual attention in conditioned fear; (3) the rate of change in V1 representation of CS+ was positively related to the rate of change in alpha ERD and (4) EEG alpha ERD activity was coupled to BOLD activity in rTPJ and to a lesser extent, rVLPFC, both of the VAN, but not to BOLD activity in the right amygdala.

### Sharpened visual representation of conditioned threat

Electrophysiological studies in humans have found visuocortical amplification of conditioned threat cues (Moratti *et al.*, 2006; Stolarova *et al.*, 2006; Miskovic and Keil, 2012; Thigpen *et al.*, 2017), accompanied by heightened inter-trial and inter-site phase locking over primary visual cortex (Keil *et al.*, 2007; McTeague *et al.*, 2015). A recent electrophysiological study in macaque monkeys also reported very early amplitude enhancement of afferent responses after aversive conditioning (Li *et al.*, 2019). The present study suggests that such changes reflect a sparsification process in the neural representation of conditioned threat, in which visual features associated with recurring, predictable threat are increasingly represented by sharpened, efficient and internally tightly coupled visuocortical networks, rather than by a generally heightened visual population response. Specifically, we found that fewer voxels contributed to the representation of CS+ as learning progressed, whereas the BOLD magnitude evoked by CS+ did not change. Sparsification of voxel patterns is conceptually consistent with notions of sharpened, efficient representations emerging as a function of Hebbian associative mechanisms. Such networks would be expected to show heightened temporal accuracy and phase stability across trials, prompting heightened average evoked responses, which is what has been observed in previous studies (Miskovic and Keil, 2012). Intracranially, highly connected and optimally tuned V1 circuits are likewise expected to produce increased population level firing via a similar mechanism when stimulated with the threat cue. This prediction is in line with recent observations in the macaque model (Li *et al.*, 2019), where CS+ gratings prompted faster multi-unit activity recorded with multi-electrode arrays, within 40 ms of stimulus onset. To what extent this increase in neural response is accompanied by sparsified multivariate neural representation, however, remains to be further explored.

Notably, the present evidence suggested that sparsification persisted throughout extinction, despite the finding that the selective HR orienting response to the CS+ was extinguished. The observation that changes in visuocortical activity are more resistant to extinction than autonomic or behavioral indices is consistent with studies on experimental animals as well as human participants (McTeague *et al.*, 2015). These studies have shown sustained sensory learning and sensory plasticity during extinction, instead of returning to a pre-conditioning, naïve, state (for a review, see McGann, 2015). Sparsification has been discussed as a key aspect of such ongoing plasticity because it minimizes metabolic cost while enabling specific and efficient representations of predictable threat cues (Miskovic and Keil, 2012; McGann, 2015).

Alternatively, a body of research has suggested that repeating visual stimuli produces neural activity reduction in the visual cortex, called repetition suppression, which is potentially accompanied by sharpened representations (e.g. Gruber and Müller, 2002; Gruber *et al.*, 2004; Delorme and Makeig, 2004). A repetition suppression effect alone is, however, unlikely to explain the present set of findings because (1) decreasing activity induced by viewing the same stimulus repeatedly has been primarily observed with familiar, meaningful objects and scenes, whereas repetition of unfamiliar stimuli devoid of rich semantics (such as the gratings used in the present study) may lead to an increase of neural activity under this perspective (Conrad *et al.*, 2007), (2) the number of repetitions of the CS+ and CS- was equal across the trial types (60 trials each), thereby ruling out the effect of uneven stimulus exposure and (3) the voxel pattern difference prompted by the two stimuli persisted during extinction training in which both CS+ and CS- were shown in identical fashion, with no US given. Furthermore, whereas the number of voxels that selectively represented the CS+ became smaller as learning progressed, the overall BOLD activity within these voxels did not change.

Another line of possible argument is that the change and sparsification of the V1 BOLD patterns may simply be a reflection of diminished engagement with the threat cue over the course of the acquisition session. The increased alpha ERD for the CS+ with learning, however, is more consistent with the notion that attention is increasingly directed to the threat cue, contradicting a selective disengagement hypothesis. The present study also did not counterbalance orientations of the Gabor patches across participants, such that the same orientation served as the CS+ for each participant. This could represent a limitation in that any systematic difference between +45° and −45° in associability or habituation/adaption would influence the current results. To the extent however that we analyzed RSA changes relative to a robust voxel pattern based on the entire habituation block, such a confound seems unlikely to drive the current findings. Together, the present data support the hypothesis that associative learning selectively shapes visuocortical representations of threat in a way that promotes sparser, sharpened coding of the critical stimulus features (Kok *et al.*, 2012; Ibrahim *et al.*, 2016).

How do the results reported here relate to the theory of sparse neural coding? In single unit neurophysiology, sparse neural coding refers to the representation of a stimulus by a small set of neurons (Simoncelli and Olshausen, 2001). It is obvious that fMRI cannot resolve neural activity at the single cell level. However, if we view the voxel as the unit of measurement and analysis, some parallels can still be drawn between multivoxel pattern analysis in fMRI and multiunit pattern analysis in single unit neurophysiology. In fact, techniques such as support vector machine (SVM) and RSA are shared between the two fields. It is interesting to note that the sparse coding hypothesis has proven to be rather difficult to test in single unit neurophysiology (Berkes *et al.*, 2009). The main obstacle is how to define ‘a small set of neurons.’ In our study, sparsification is defined in the context of learning, and inferred from the comparison between the early and late periods of acquisition. It is quite clear that, without such comparison, the notion of sparsity in fMRI will also be difficult to define.

### Defensive orienting and EEG alpha ERD

Previous work has shown that the cardiac orienting response to threat, measured as phasic HR deceleration when viewing the CS+, is attenuated as learning progresses (Sokolov, 1963; Bradley, 2009; Yin *et al.*, 2018). This adaptation in HR orienting is concomitant with adaptation in canonical fear circuits and the salience network, including the amygdaloid complex, dorsal anterior cingulate cortex and anterior insula (Yin *et al.*, 2018). By contrast, in the present study, EEG alpha ERD—a phenomenon associated with visual activation and attentive stimulus processing—became stronger (increased sensitization) during the course of acquisition. This is consistent with the long-held notion that behavioral, autonomic and neurophysiological responses to threat are not linearly related (Lang, 1979), reflective of their different adaptive functions in addressing the threat.

A large body of research has shown that the extent of event-related alpha power reduction or alpha ERD over visual areas co-varies with the motivational significance (task-relevance) and/or perceptual saliency of the event (Ruby *et al.*, 2013). Thus, the present finding that alpha ERD becomes stronger with conditioning suggests that the selective/attentive processing of the CS+ is increasing, not decreasing, as learning progresses. Supporting this interpretation, a previous study found the adaptation of limbic brain areas to be accompanied by increased engagement of visual cortex during fear conditioning (Lithari *et al.*, 2016). Such persistent visuocortical engagement with the threat cue may be particularly adaptive in conditioning regimes with intermittent pairing, in which not all CS+ trials include a US presentation, promoting exploration behavior and scanning of the environment for contingency cues—a hypothesis that is readily testable in future research and consistent with extinction-resistant alpha power changes during a 2-day conditioning regimen (Panitz *et al.*, 2019). Notably, heightened attention to the threat cue is unlikely to explain the cross-species observation that extensive conditioning over time prompts selectively heightened visuocortical responses at very short latencies and in retinotopic visual areas. The present findings are, however, consistent with earlier work that has emphasized the role of heightened top-down signaling in selective threat cue processing (Petro *et al.*, 2017), as plastic changes mediated by attention selection history can alter the sensitivity of retinotopic neurons (cf., Li *et al.*, 2019).

### Sources of modulatory signals mediating visuocortical changes

Most contemporary viewpoints agree that heightened visuocortical responses result from interactions between visual and extra-visual brain regions, with the latter conveying modulatory signals that selectively heighten the gain of visual neurons, individually or at the population level. Two candidate circuits for providing such re-entrant modulatory feedback to visual cortex have received the most attention in the literature: the amygdala and the VAN. Two mechanisms have been proposed for amygdalofugal modulations of the visual system. One is through its projections to earlier levels of the visual pathway including primary and secondary visual cortices to enhance perceptual processing of emotional stimuli (Amaral *et al.*, 2003). The other is through its connections with higher order attentional modulation areas such as the intraparietal sulcus (Armony and Dolan, 2002) and VLPFC (Ghashghaei *et al.*, 2007). Consistent with earlier work testing the amygdalofugal re-entry hypothesis in fear conditioning (Petro *et al.*, 2017), the present study did not find support for the notion that hemodynamic activity in the amygdaloid complex co-varies with selective visuocortical processing of the CS+, neither at the level of BOLD nor at the level of scalp-recorded electrophysiology. Targeted studies in the animal model are needed to characterize the role of the amygdala in biasing visuocortical processes during fear conditioning.

The VAN consisting of rTPJ and rVLPFC is thought to mediate the allocation of attention in response to the presence of salient sensory stimuli (Fox *et al.*, 2006; Vossel *et al.*, 2014). For example, BOLD activity in areas within the VAN such as the rTPJ is modulated by tasks that require participants to selectively attend to events varying in hedonic valence and/or arousal (Fichtenholtz *et al.*, 2004; Lee and Siegle, 2012; Klimesch, 2012). Although not suitable for establishing causality, the present findings support the hypothesis (Petro *et al.*, 2017) that, even in the absence of a cognitive task, biasing signals originating in attention-related brain regions such as rTPJ facilitate the selective visuocortical processing of conditioned threat cues. Further illustrating a dissociation of limbic and attention networks, competing macroscopic networks may be active during different phases of classical fear conditioning, with limbic and prefrontal networks being anti-correlated (Marstaller *et al.*, 2016). Future work may address the extent to which VAN engagement in fear acquisition is driven by input from threat-modulated regions such as the amygdala or insula.

### Summary and conclusions

The present study showed that extensive fear conditioning prompts the emergence of sharpened, sparser pattern representations of the condition threat in visual cortex. These pattern changes were characterized by decreasing numbers of voxels showing CS+ specificity. The rate of CS+ representational pattern changes co-varied with the rate of increased CS+ evoked alpha ERD, with alpha ERD being associated with the activity of VAN, rather than the amygdala. The sparsification of voxel patterns persisted during extinction training, in line with electrophysiological work showing lasting changes in afferent visuocortical processing after extensive fear conditioning (Thigpen *et al.*, 2017), despite the fact that autonomic responses to CS+ and CS- showed no difference. Together, these observations support the notion that sustained fear learning prompts plastic changes at the lowest level of visuocortical processing stream to cope with the demands posed by an ever-changing environment, and to facilitate the detection and identification of threats or opportunities, and that attention mechanisms play a significant role in this process.
